# Effect of Mobile-Based Lifestyle Intervention on Body Weight, Glucose and Lipid Metabolism among the Overweight and Obese Elderly Population in China: A Randomized Controlled Trial Protocol

**DOI:** 10.3390/ijerph18094854

**Published:** 2021-05-01

**Authors:** Yu Zhang, Xiaohui Guo, Na Zhang, Xinyu Yan, Muxia Li, Mingzhu Zhou, Hairong He, Yibin Li, Wen Guo, Man Zhang, Jianfen Zhang, Guansheng Ma

**Affiliations:** 1 Department of Nutrition and Food Hygiene, School of Public Health, Peking University, Beijing 100191, China; zhangyu30171026@163.com (Y.Z.); ziqingxuanping@126.com (N.Z.); hilaryyxy@bjmu.edu.cn (X.Y.); lmuxia91@126.com (M.L.); zmz6290@163.com (M.Z.); hehairong_16@bjmu.edu.cn (H.H.); 1310306130@bjmu.edu.cn (Y.L.); wguo14@pku.edu.cn (W.G.); zhangman@bjmu.edu.cn (M.Z.); 13161522166@163.com (J.Z.); 2 Laboratory of Toxicological Research and Risk Assessment for Food Safety, Peking University, Beijing 100191, China; 3 College of Food Science and Nutritional Engineering, China Agricultural University, Beijing 100083, China; guoxiaohui@cau.edu.cn

**Keywords:** overweight and obesity, mobile-based lifestyle intervention, elderly population, body weight, glucose and lipid metabolism

## Abstract

*Background*: Promotion of a healthy lifestyle is considered a good strategy for dealing with chronic diseases. Mobile-based lifestyle interventions have shown beneficial effects in the control and treatment of chronic diseases such as diabetes, obesity and metabolic syndrome. Current clinical trials for mobile-based lifestyle intervention were mainly conducted among non-elderly populations, thus well-designed trials performed among the elderly who are more susceptible to chronic diseases are needed. The study aims to assess the effect of the mobile-based lifestyle intervention on the improvement of body weight, glucose and lipid metabolism among overweight and obese elderly adults in China. *Materials and Methods*: Participants aged 60–80 years who are overweight or obese will be randomly assigned to receive mobile-based nutrition and exercise intervention, mobile-based exercise intervention and no intervention for 3 months. Before the intervention, participants will receive the training of the mobile application and sports bracelet. The primary outcome will be the between-group (three groups) difference in body mass index at the end of intervention. The secondary outcomes will include body composition, parameters of glucose and lipid metabolism, blood pressure, dietary data and physical activity data. All these outcomes will be assessed at baseline, day 45 and day 90. *Ethics and dissemination*: The trial has been approved by the Ethics Committee of Peking University Health Science Center (IRB00001052-18039).

## 1. Introduction

Overweight and obesity have increased globally and substantially in recent years [1]. In China, the prevalence of overweight and obesity increased from 37.4% and 8.6% to 41.2% and 12.9%, respectively, which has become a major public health problem [2]. These conditions are proved to be associated with chronic diseases such as hypertension, diabetes, non-alcoholic fatty liver disease, coronary artery disease and dyslipidemia and cause a reduction of life expectancy [3]. 

Aging is also a risk factor for many chronic diseases, due to the decline of physiological functions in the elderly. Chronic diseases have become a great health threat to elderly residents in China. The prevalence rates of hypertension, diabetes and hypercholesterolemia in China were 58.3%, 19.4% and 10.5%, respectively, among people older than 60 years old, which were higher than that of people less than 60 [4]. According to the data from the National Bureau of Statistics, the number of Chinese people aged 65 or over in 2019 was 176 million, which represents 12.6% of the whole population of China. With the increasing aging population, the healthy and economic burden created by chronic diseases can be expected to become more and more serious. 

These chronic diseases which are major causes of morbidity and mortality have been considered to be closely related to changes in dietary and lifestyle [5]. As a result, the promotion of a healthy lifestyle has been seen as a key strategy of reducing such chronic diseases [3]. A lot of studies have demonstrated that lifestyle intervention has a beneficial effect on reducing the diabetes incidence among impaired glucose tolerance patients [6] and improvement of obesity [7], hypercholesterolemia [8], hypertension [9], non-alcoholic fatty liver disease [10] and metabolic syndrome [11]. However, in the traditional types of lifestyle intervention programs, patients usually need to communicate with a clinician or qualified nutritionist in community health centers or physician’s offices, thus the traditional lifestyle intervention through in-person education programs is resource intensive [12]. In addition, the effect of traditional lifestyle intervention decreases or even disappears after several years [13], which may be because it is hard to change people’s lifestyle [6]. 

Advances in mobile technology make it possible to supply health management support to patients which is both convenient and cost-effective [12]. Mobile applications (apps) for health management have been shown to play a positive role in improving several chronic diseases and may bring enormous social and economic benefits. Kumar et al. found that mobile-based lifestyle intervention could decrease the hemoglobin A1c level of Type 2 diabetes patients significantly [14]. An American study conducted among patients with obesity, hypertension, diabetes and hyperlipidemia showed that mobile-based interventions produced larger weight loss compared with the control group during the whole 12-month follow-up [15]. Another study demonstrated that overweight and obesity Korean patients, after following a mobile-based lifestyle intervention, had a clinically significant weight loss and maintained this effect during the 52-week follow-up and also showed significant improvement in metabolic syndrome parameters such as fasting glucose levels and blood lipids [16]. 

The overweight and obese elderly populations with two risk factors, aging and high-BMI, are more susceptible to chronic diseases. However, most clinical trials for mobile-based lifestyle intervention were conducted among non-elderly populations [14,15,16,17]. Considering the data on the effect of mobile-based lifestyle intervention on the control of chronic diseases among the elderly population was very limited, well-designed randomized controlled trials (RCTs) performed in the elderly are needed. 

The purposes of this study are, first, to assess the effect of the mobile-based lifestyle intervention on the improvement of body weight, glucose and lipid metabolism among overweight and obese elderly adults in China. In addition, to explore whether the mobile-based lifestyle intervention may have an effect on blood pressure, body composition, C-reactive protein, liver enzymes, uric acid and the factors associated with blood glucose or lipid including copeptin, irisin and adiponectin. Third, to compare differences between males and females. Finally, to provide suggestions for the intelligent health management among the elderly in China.

## 2. Materials and Methods

### 2.1. Sample Selected

The recruitment of the study subjects will take place in communities through advertisements in five cities (Jinan, Taiyuan, Nanchang, Hefei, and Guangzhou) in China. After recruitment, basic information such as social demographics, disease history, drug use and family history will be collected.

Subjects eligible for this trial must meet the following inclusion criteria: (1) age between 60–80 years old; (2) body mass index (BMI) ≥ 24; (3) living or working in Jinan, Taiyuan, Hefei, Nanchang and Guangzhou and (4) ability to use smart phones. The exclusion criteria will include: (1) participation in any activities related to weight management; (2) diagnosed with psychiatric disorder such as cognitive dysfunction, schizophrenia or depression; (3) with pacemakers or other medical electronic devices; (4) with walking disability; (5) history of surgical treatment of obesity; (6) excessive drinking and (7) unwillingness to change their habits. 

### 2.2. Study Design

**Figure 1:**
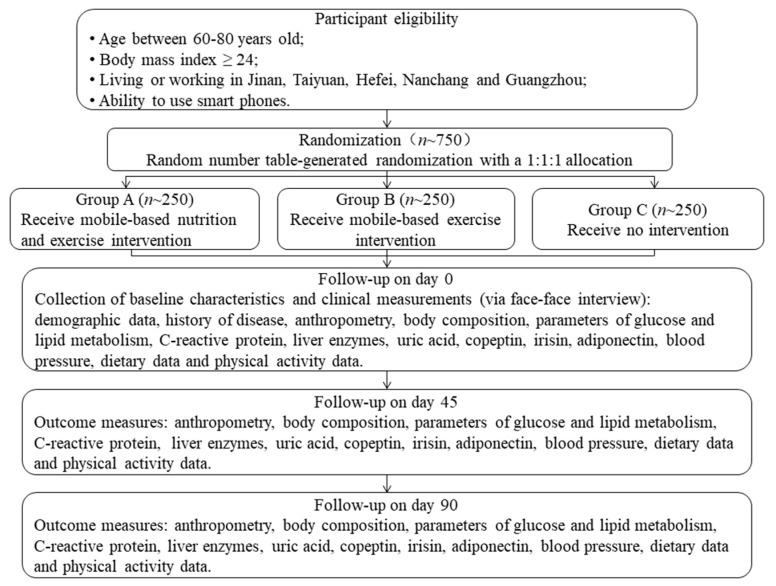
The flow diagram of this trial.

This study is designed as a prospective, open-labeled, randomized, controlled and multi-center trial. Eligible subjects will be randomly assigned to three groups: the mobile-based nutrition and exercise intervention group (Group A), mobile-based exercise intervention group (Group B) and no intervention group (Group C). This study will assess parameters of glucose and lipid metabolism for three times. In addition, weight, height, waist circumference, hip circumference, blood pressure, C-reactive protein, liver enzymes, uric acid, copeptin, irisin, adiponectin, dietary data and physical activity data will also be evaluated at the same time. The study design is shown in Figure 1.

### 2.3. Randomization and Blinding

All participants will be assigned to three groups according to a random number table with a 1:1:1 allocation ratio. The assignment will be enclosed in sealed, opaque, numbered envelopes by an investigator not involved in subject assessment and recruitment. Because of the nature of the intervention, it is not possible to blind participants and study investigators to the group allocation. To minimize bias, at every visit, outcome assessors will be blinded to group allocation and objective clinical measures will be measured. Investigators who analyses the data will also be blinded to the data collection and data entry, as each subject will be identified by a random number.

### 2.4. Intervention

Volunteers allocated to Group A and B will have access to a health management app. The app has functions of recording and monitoring the participants’ diet and exercise daily. The interventions include mobile-based nutrition interventions and exercise interventions. 

The nutrition interventions require the subjects be provided with 20 g of the professional meal replacement powders (Huaruizhongjian Biotechnological Co., Ltd., Shandong, China), 200 mL milk and 5 mL flaxseed oil for their breakfast and dietary guidance based on Mediterranean diet for their lunch and supper. The meal replacement powders include 10 g of meal replacement powders rich in minerals (4.50 g protein, 0.09 g fat, 1.26 g carbohydrate and 3.00 g dietary fiber with 125 kJ in total energy) and 10 g of meal replacement powders rich in vitamins (4.65 g protein, 0.11 g fat, 1.05 g carbohydrate and 3.18 g dietary fiber with 126 kJ in total energy). The dietary guidance will be through individual sessions with nutritionists using mobiles, including healthy food choices and portion sizes, 3 to 5 times per week depending on the participants’ compliance. The diets will be tracked and recorded through uploading the food logs and pictures of their three meals per day using the health management app under the supervision of the investigators in the background. 

The exercise interventions require the subjects be provided with exercise guidance and complete around 20 min of aerobic exercise, 20 min of resistance exercise and at least 6000 steps every day. The exercise guidance will be through individual sessions with exercise instructors, including exercise choices, duration and intensity, 3 to 5 times per week depending on the participants’ compliance. If the participants feel a little tired after the exercise and comfortable for the next day, the exercise intensity will be the best. The everyday steps of the subjects will be tracked and recorded through the sports bracelet and the aerobic exercise and resistance exercise logs and pictures will be uploaded using the mobile app, which will be supervised by the investigators in the background. 

Participants in Group A will receive nutrition interventions and exercise interventions for three months. Group B will receive exercise interventions for three months and Group C, the control group, will not be intervened and expected to follow their routine lifestyle as before. Interventions will be started one week after the enrollment.

The app and sports bracelet training will be provided in the same manner for both Group A and Group B volunteers one week before the start of this trial. The researchers will help the participants download the health management app through the smart phone app store and teach them to use the app and the sports bracelet. After the participants have learnt how to use the app and the bracelet, they will be asked to record their dietary data and physical exercise intensity following their old way of life for one week before the start of the intervention using the app and the bracelet, to guarantee that they have already grasped the usage of the app and the bracelet. As this is lifestyle intervention, the medication for the baseline chronic diseases will not be changed during the course of the study.

### 2.5. Follow-Up

All participants of this study will be followed-up for the duration of the intervention (three months). Participants may discontinue the trial at their request. If a participant does not meet the requirements of the study, he should also discontinue the trial. 

### 2.6. Procedures

A summary of this trial is presented in Table 1. The study will consist of three stages:

Stage 1: from enrollment to the start of the intervention. At enrollment, eligible participants will sign informed contents and their basic information will be collected. Then they will be randomly divided into three groups (Group A, B and C). Participants in Group A and B will be taught how to use the app and sports bracelet by the researchers and required to record their diets and physical activities using the app and bracelet following their usual way of life for one week.

Stage 2: from the start of intervention (day 0) to day 44. On the morning of day 0, participants will be asked to arrive at the lab at 7:00 a.m. Measurements including anthropometry, body composition, blood pressure, fasting blood samples, dietary and physical activity data will be obtained based on standard procedures. Then, participants in Group A will receive mobile-based nutrition and exercise intervention. Participants in Group B will receive mobile-based exercise intervention. Participants in Group C will receive no interventions.

Stage 3: from day 45 to day 90. On the morning of day 45, measurements including anthropometry, body composition, blood pressure, fasting blood samples, dietary and physical activity data will be performed as day 0. Then, Group A and B will continue to receive their respective intervention and Group C will receive no intervention as before. On the morning of day 90, anthropometry, body composition, blood pressure, dietary and physical activity data will be measured and fasting blood samples will be collected for the third time. 

**Table 1:** Summary of the trial.

Time Point	At Enrollment	After Allocation		
		Start (Day 0)	Day 45	Day 90
Enrolment				
Eligibility screen	√			
Informed consent	√			
Collection of basic information	√			
Randomization	√			
App and sports bracelet training	√			
Interventions (day 0-day 90)		√	√	√
Follow-up (day 0-day 90)		√	√	√
Assessments				
Anthropometry	√	√	√	√
Body composition (Inbody measurement)		√	√	√
Blood lipids		√	√	√
Fasting and postprandial blood sugar		√	√	√
Hemoglobin A1C		√	√	√
Blood CRP and insulin		√	√	√
Blood ALT and AST		√	√	√
Blood uric acid		√	√	√
Blood copeptin		√	√	√
Blood irisin		√	√	√
Blood adiponectin		√	√	√
Blood pressure		√	√	√
Dietary data		√	√	√
Physical activity data		√	√	√

CRP: C-reactive protein; ALT: alanine aminotransferase; AST: aspartic transaminase.

### 2.7. Outcomes

The primary outcome and secondary outcomes are presented in Table 2.

#### 2.7.1. Anthropometry

Body weight and height will be obtained at enrollment and every study visit. Body weight (kg) will be measured twice to the nearest 0.1 kg without shoes, in light indoor clothing, and standing height (cm) will be measured twice to the nearest 0.1 cm, barefoot and the head positioned in the Frankfurt horizontal plane by trained investigators using a height-weight meter (HDM-300; Huaju, Zhejiang, China) [18,19]. All participants will be asked to visit a toilet before the measurements. Overweight will be defined as 24 ≤ BMI < 28 kg/m^2^ and obesity will be defined as BMI ≥ 28 kg/m^2^ [20].

#### 2.7.2. Body Composition 

Body composition parameters include body fat mass (BFM), visceral fat area (VFA), fat free mass (FFM), body fat percent (BFP).

This outcome will be assessed with multi-frequency bioelectrical impedance analysis with 8-point tactile electrodes (InBody 720; Biospace, Seoul, Korea) [21]. Bioelectrical impedance will be measured within 2 min, standing on bare foot and grasping the hand electrodes with arms in the vertical position as is described in an article of our team [22]. Body composition will be measured at baseline, day 45 and day 90.

#### 2.7.3. Parameters of Glucose and Lipid Metabolism, C-Reactive Protein, Liver Enzymes, Uric Acid, Copeptin, Irisin and Adiponectin

These parameters will be obtained at baseline, day 45 and day 90. Serum lipids [triglyceride (TG), total cholesterol (TC), high-density lipoprotein cholesterol (HDL-C), low-density lipoprotein cholesterol (LDL-C) (mg/dL)], fasting plasma glucose (FPG), hemoglobin A1C, serum uric acid, copeptin, irisin, adiponectin, insulin and C-reactive protein will be measured after an overnight fast and 2 h postprandial plasma glucose (2 hPPG) will be measured 2 h after the breakfasts at the baseline visit, day 45 and day 90 in all participants. Plasma glucose will be measured by hexokinase method. Serum TC, HDL-C and LDL-C will be measured using an enzymatic colorimetry method. Serum TG and uric acid concentrations will be determined by colorimetric. Serum ALT and AST will be measured using International Federation of Clinical Chemistry method. Serum copeptin, irisin and adiponectin will be measured with enzyme-linked immunosorbent assay. Blood hemoglobin A1C will be measured using high performance liquid chromatography. Serum insulin will be measured using chemiluminescent immunoassay and C-reactive protein will be determined by immunoturbidimetry.

Dyslipidemia will be defined as self-reported history of dyslipidemia or levels of TC ≥ 6.2 mmol/L, LDL-C ≥ 4.1 mmol/L, TG ≥ 2.3 mmol/L, or HDL-C less than 1.0 mmol/L [23]. Impaired FPG (IFG) will be defined as 6.1 ≤ FPG < 7.0 and 2 hPPG < 7.8, and IGT will be defined as FPG < 7.0 and 7.8 ≤ 2 hPPG < 11.1. Diabetes will be defined as self-reported history of diabetes or FPG ≥ 7.0 mmol/L or 2 h plasma glucose ≥ 11.1 mmol/L on 75 g oral glucose tolerance test [24]. Elevation of serum uric acid will be defined as serum uric acid level > 420 μ-mol/L after consumption of a normal daily diet [25]. 

#### 2.7.4. Blood Pressure (Systolic and Diastolic)

Blood pressure of right upper limb will be measured in the sitting position at each study visit using a validated semiautomatic sphygmomanometer (Omron HEM-705CP, Dalian, China) by trained nurses. Two measurements will be conducted at interval of at least 3 min, and the average of two readings will be used for analysis. If the difference between the first two readings is ≥5 mm Hg, additional (one or two) readings will be obtained. 

Hypertension is defined as self-reported history of hypertension or systolic blood pressure (SBP) higher than 140 mmHg or diastolic blood pressure (DBP) higher than 90 mmHg [26].

#### 2.7.5. Dietary Data and Physical Activity Data

Dietary data and physical activity data will be measured at baseline, day 45 and day 90. Dietary data assessment will be based on the Food Frequency Questionnaire (FFQ), dietary logs and pictures on the app. The type, amount and frequency of food intake will be recorded by trained investigators. The amount of food will be estimated using the references of food models or photographic models [27]. Nutrients intake will be assessed using the Chinese Food Composition Table [28]. Physical activity will be assessed through the exercise logs, pictures and the average steps of 3 days before the baseline visit, day 45 and day 90 using the sports bracelet.

### 2.8. Sample Size Calculation

The sample size was calculated for a one-way analysis of variance (ANOVA) comparing the mean change in between-group (three groups) BMI from preintervention to postintervention. According to related researches, the clinically meaningful difference in change in BMI was set to 1 kg/m^2^ and the mean change in BMI of the 12-week diet and exercise group, the 12-week exercise group and the control group was −2.8, −2.5 and −0.9 kg/m^2^, respectively. The standard deviation (SD) was assumed to be equal for all three groups and was set to SD = 1.4 Kg/m^2^ [29,30]. To obtain a power of 0.90 for the overall test of differences in means, using a significance level of 0.05, 13 individuals are required in each group. To account for the three stratification groups (age: 60~66, 67~73 and 74~80), a further 26 individuals are required. Considering the study will be conducted in 5 centers, approximately 200 individuals are required for each group. To account for 20% dropout, approximately a total of 250 individuals are required for each group. Calculations were performed using PASS statistical software Version 15.0.2 (NCSS, Kaysville, UT, USA).

### 2.9. Statistical Analysis

Continuous data will be first tested for normal distribution using the Kolmogorov-Smirnov test and presented as mean ± standard deviation (SD) if normally distributed, or median [interquartile range (IQR)] if not. Categorical data will be presented as percentages.

The effect of mobile-based lifestyle intervention on BMI, parameters of glucose and lipid metabolism, body composition, C-reactive protein, liver enzymes, uric acid, copeptin, irisin, adiponectin and blood pressure among the three groups will be investigated by One-way ANOVA or Kruskal-Wallis H test. 

*p* values will be two-tailed and differences will be considered significant at *p* < 0.05. All analyses will be performed using SPSS software Version 23.0 (SPSS Inc., Chicago, IL, USA).

### 2.10. Ethics

The protocol and template consent forms of this study have been approved by the Ethics Committee of Peking University Health Science Center (IRB00001052-18039). This study will adhere to the rules of the Declaration of Helsinki. Written informed consent will be obtained from all participants. Any modifications to the protocol, which may affect the conduct of the study or the participants’ benefits or safety, will be reported to the ethics committee. All information related to this study will be stored securely at School of Public Health of Peking University, in an area with limited access. Any information collected in this project will not be used for any other purpose. The investigators and associated research personnel will have access to information. 

### 2.11. Providing Suggestions

Suggestions for intelligent health management among the elderly in China will be provided. As the elderly have their own physical and mental characteristics and knowledge structure, recommendations on how to improve compliance, how to make the health management app more friendly, how to improve ease of use, how to create incentives, how to make the health knowledge and healthy lifestyle more acceptable to the elderly will be made based on the experiences during the conduction of the study. And if the result proves to be that the mobile-based lifestyle intervention is beneficial, it would provide a successful approach for the improvement of bodyweight, glucose and lipid metabolism and a scientific evidence for intelligent health management among the elderly in China.

### 2.12. Dissemination

The findings of this study will be disseminated via peer-reviewed publications specialized in nutrition or public health or presentations in relevant national and international conferences. The results will be available to the participants on request at a face-to-face meeting.

## 3. Trial Registration

The clinical trial registration number is ChiCTR1900023355 (http://www.chictr.org.cn/showproj.aspx?proj=38976). Date of registration: 23 May 2019.

## 4. Discussion

Overweight and obesity is associated with chronic diseases such as hypertension, diabetes and cardiovascular diseases [31]. These chronic diseases, as a main threat to health, led to three-quarters of deaths in China [32]. They have been considered to be closely influenced by unhealthy lifestyle [5], thus promotion of healthy lifestyles is a key strategy for reducing chronic disease risk [33]. Compared with traditional lifestyle intervention which is resource intensive and the effect decreases as time goes on [13,34], the new mobile-based lifestyle intervention, with its own advantages, is an attractive option for the prevention and control of chronic diseases. It has been shown that mobile-based lifestyle intervention is effective in the improvement of abnormal glucose and lipid metabolism and the control of obesity in several previous studies [16,17,35]. China has already entered an aging society phase with a large proportion of elderly people suffering from chronic diseases [36]. However, the effectiveness of mobile-based lifestyle intervention for the control of overweight, abnormal glucose and lipid metabolism among elderly population with a predisposition to chronic diseases is still unclear. This study intends to fill a gap in this field. 

The compliance of the subjects and the quality control will be the challenges during the study. Subjects in Group A and B will be asked to be intervened for as long as 3 months. Moreover, the elderly population whose learning ability to use mobile apps is not as good as the young may face more challenges. Poor utilization of app may cause the reduced effect of intervention [12]. In order to guarantee the compliance of them, convenient and effective methods of communication, such as telephone call, WeChat, short message and e-mail, between investigators and participants will be established. The investigators will fully explain the requirements and significance of this study to the subjects prior to the study, monitor their dietary and physical activity data and solve the problems they encounter in time. If a subject quits, the investigators will record the time and reasons timely. To ensure the quality control, participants will be trained to be familiarized with the content of questionnaires, the procedure and requirements of the trial prior to the study. If a participant fails to meet the requirements, their data will be eliminated and the reason will be recorded. Researchers will also be trained on the procedure of this trial, the content of the questionnaires and the requirements of laboratory tests. Indicators such as height, weight, waist circumference, hip circumference and blood pressure will be measured twice and the mean will be adopted as the final outcome. When filling in the FFQ, food models and photographic models will be used to enhance estimation accuracy. Blood samples will be stored at −80 degree centigrade immediately after collection to ensure the accuracy of laboratory tests. All laboratory measures will be operated by experienced staff. The conduct of this trial will be audited by a competent authority in the university every week, and the process will be independent from investigators and the sponsor. 

This study has both strengths and limitations. In terms of strengths, it will be designed as a randomized controlled trial which can reduce the selection bias and confounding influence. The consistence of baseline characteristics between groups will guarantee that the three groups are comparable. Secondly, the potential confounding factors such as age, sex, marital status, literacy, occupation, the state of chronic diseases, food intake, physical activity, living conditions and family and social support will be evaluated and included as covariates in the statistical analyses. What’s more, the prospective cohort design can avoid recall bias and improve the accuracy of the results. Fourthly, the sample size calculated will provide sufficient power to detect the effects. Fifthly, outcomes will be measured three times during the intervention and follow-up which will supply more detailed data. Finally, this will be a large multi-center study. Compared with the previous single-center study, our study will represent a more comprehensive and realistic assessment of the mobile-based lifestyle intervention effect among elderly population. In terms of limitations, considering the cost and feasibility, a 3-month intervention will be conducted which is short and the long-term effect may not be demonstrated. However, our finding will provide a preliminary data for the future studies with a longer-term intervention and follow-up. In addition, this study is designed as an open-labeled RCT because of the nature of the intervention which may cause bias. However, the outcome assessors and data analysts will be blinded to the group allocation and objective measures will be used as outcomes to minimize the bias. The Hawthorne effect will be avoided as much as possible through paying equal attention to the three groups.

## 5. Conclusions

In conclusion, although the mobile-based lifestyle intervention is an attractive option for health improvement, evidence in the elderly population who are susceptible for chronic diseases still needs to be verified. It will be the first time the effectiveness of mobile-based lifestyle intervention for reducing body weight and improving glucose and lipid metabolism in overweight and obese elderly population in China is tested. Our results will provide evidence for promoting mobile-based healthy lifestyle intervention and the prevention and control of chronic diseases among elderly population in China. It will also raise the public’s awareness on the importance of promoting a healthy lifestyle.

## Figures and Tables

**Table 2:** Primary and secondary outcomes.

Outcomes	Assessments
Primary outcome	the between-group (three groups) difference in body mass index at the end of intervention
Secondary outcomes	parameters of glucose and lipid metabolism, CRP, liver enzymes, uric acid, copeptin, irisin, adiponectin, body composition, blood pressure, dietary data and physical activity data

CRP: C-reactive protein.
